# B-Site Nanoscale-Ordered Structure Enables Ultra-High Tunable Performance

**DOI:** 10.34133/2022/9764976

**Published:** 2022-10-26

**Authors:** Biaolin Peng, Qiuping Lu, Yi-Chi Wang, Jing-Feng Li, Qi Zhang, Haitao Huang, Laijun Liu, Chao Li, Limei Zheng, Zhong Lin Wang

**Affiliations:** ^1^School of Advanced Materials and Nanotechnology, Xidian University, Xi'an 710126, China; ^2^Beijing Institute of Nanoenergy and Nanosystems, Chinese Academy of Sciences, Beijing 101400, China; ^3^State Key Laboratory of New Ceramics and Fine Processing, Department of Materials Science and Engineering, Tsinghua University, Beijing 100084, China; ^4^Basque Centre for Materials, Applications and Nanostructures, UPV/EHU Science Park, Barrio Sarriena s/n, 48940 Leioa, Spain; ^5^Ikerbasque, Basque Foundation for Science, Plaza Euskadi, 5, Bilbao 48009, Spain; ^6^Department of Applied Physics, The Hong Kong Polytechnic University, Kowloon, Hong Kong; ^7^Guangxi Key Laboratory of Optical and Electronic Materials and Devices, Guilin University of Technology, Guilin 541004, China; ^8^Instrument Analysis Center, Xi'an Jiaotong University, Xi'an 710049, China; ^9^School of Physics, Shandong University, Jinan 250100, China; ^10^School of Materials Science and Engineering, Georgia Institute of Technology, Atlanta, Georgia 30332-0245, USA

## Abstract

Tunable devices constructed by ferroelectric thin films are often desired to possess a low dielectric loss while maintainging a high dielectric tunability over a broad operating temperature range in applications, for example, resonators, filters, or phase shifters. However, it is difficult to simultaneously achieve these characteristics by traditional strategies, such as doping and strain modifying. Here, we demonstrate that the dielectric tunability of the sol-gel-prepared Pb(Sc_1/2_Nb_1/2_)_0.9_(Mg_1/3_Nb_2/3_)_0.1_O_3_ (PSNMN) thin film can be almost doubled from ~47% to ~80.0% (at 10 kHz) at a low electric field (~530 kV/cm), and the dielectric loss can be sharply reduced by more than an order of magnitude, from ~0.50 to ~0.037 (at 1 kHz) when the thin film was annealed in air at 650°C for 15 h under the help of an atmosphere-compensating-block (ACB) made from the proto-PSNMN gel. Moreover, the PSNMN thin film annealed with ACB also exhibited an extremely high thermally-stable dielectric tunability in an ultrabroad temperature range (>130 K), which could be attributed to the Maxwell-Wagner (MW) effect generated by the interface between the PSNMN disordered matrix and the B-site nanoscale-ordered structure formed during the long-term annealing process. The reduced dielectric loss is mainly benefited from the reduced concentration of oxygen vacancy and the possible MW effects, and the enhanced dielectric tunability could be ascribed to the weaker domain-pinning effect by oxygen vacancy. The breakthrough provides a new universal strategy to achieve utrahigh tunable performance in A(B'_1/2_B”_1/2_)O_3_ ferroelectric thin films with a B-site nanoscale-ordered structure, meanwhile it paves the way for ultraintergrated tunable thin-film-devices with great phase shifter performance in practical applications.

## 1. Introduction

With the rapid development of electronic information technology, highly tunable devices, such as resonators, filters, or phase shifters, have been increasely demanded by the civil and military communication systems such as radars and 5G/6G mobile communications. However, traditional tunable devices based on the ferrites and semiconductor PIN diodes can no longer meet the requirements of high phase shift accuracy, low insertion loss, wide frequency band, high reliability, light weight and small size, etc. Ferroelectrics as alternative tunable materials are very likely to satisfy these requirements, and tunable devices developed by ferroelectric thin films would be featured with a simpler structure, a higher tunability, a higher sensitivity, and a lower operable-electric-field, etc. A great number of research works based on dielectric tunability of ferroelectric thin films have been reported, and they are mainly focused on the Ba_(1-x)_Sr_x_TiO_3_, BaZr_x_Ti_(1-x)_O_3_, Pb_(1-x)_Sr_x_TiO_3_, and other inorganic titanium-containing perovskite materials. In addition to the perovskite thin films, some nonperovskite thin films were also reported, such as the hafnium oxide- (HfO_2_-) based thin film with a fluorite structure [1–3]. It is a little disappointing that high dielectric tunability (*η*) and low dielectric loss (tan *δ*) of these materials are always difficult to be achieved at the same time.

Traditional strategies that balance benefits of higher dielectric tunability and lower dielectric loss include mainly (a) the element doping and (b) the second phase recombination. The former strategy includes the equivalent substitution (Zr^4+^, Sn^4+^, etc.) and the heterovalent doping (Mn^2+/3+^, Fe^2+/3+^, etc.) [4]. The equivalent substitution manipulates materials' phase transition temperature, thereby adjusting the dielectric tunability and the dielectric loss. The heterovalent doping tends to reduce the insulation of the doped material and deteriorate the electric field breakdown strength. The later strategy involves the second phase recombination, which mainly lowers the dielectric loss, and this strategy often consists the low loss oxides of magnesium and aluminum MgO, Al_2_O_3_, MgAl_2_O_4_, etc. In addition to these strategies, buffer and/or seed layer emplanting [5–8], thickness optimizing [9–11], and defect mitigating via atomic engineering [12] are also developed as strain modifying strategies to improve the dielectric tunable properties.

For practical applications, high operating temperature is a cruel reality that a tunable device has to endure because the temperature will increase with the continuous accumulation of the heat generated by the tunable materials during the repeated switching process of the applied direct current (DC) electric field. Strong packing is a conventional method to isolate the tunable devices from its high temperature surroundings. On the other hand, advanced algorithms with temperature probes are used to prevent the overheating of the device while matintaining high performance. However, these methods often increase the volume of the overall tunable device with additional processing complicacy and costs. Therefore, the improvement of the thermal stability of tunable materials would be a more engineerable strategy, which are previously demonstrated by constructing a diffused phase transition via composition-gradient-structure [13] and elements doping [14], etc. For example, compostionaly-graded Ba_(1-x)_SrxTiO_3_ thin film was reported with a high dielectric tunability (>70% across a range of 300 K) and a small temperature dependence (deviated in 13% over 500 K) [13]. A Nb-doped SrTiO_3_ single crystal substrate with a highly (111) oriented PMN-PT relaxor (*γ* ~1.81) thin film also demonstrated a high dielectric tunability (~67.5%) and a negligible variation (<2% in the temperature range from 300 K to 450 K) [14]. These methods can improve the temperature stability of the material to a certain extent, but the overall tunable performance is still much lower than expected. Moreover, high reliability is also an indispensable requirement for tunable devices. Epitaxial 0.5Ba(Zr_0.2_Ti_0.8_)O_3_-0.5(Ba_0.7_Ca_0.3_)TiO_3_ (BZT-BCT) thin film possessing a high dielectric tunability (*η* ~85%) at room temperature was reported, but the high DC electric field (~2400 kV/cm) applied on the mater ials implying a high risk of breakdown [15]. Aforementioned limitations on material engineering demand new strategies to be developed and new mechanisms to be revealed for a desirable tunable performance. Previous research work shows that B-site ordering ceramic with A(B'_1/2_B”_1/2_)O_3_ perovskite type structure exhibits good dielectric tunable overall properties [16]. However, as far as we know, the dielectric tunability was hardly reported for the thin-film-structure of this type of material. For the Pb(Sc_1/2_Nb_1/2_)_x_(Mg_1/3_Nb_2/3_)_(1-x)_O_3_ bulk ceramic system, it is easier to obtain a B-site ordered structure with the increase of (Sc_1/2_Nb_1/2_) content, and the size of the B-site ordered structure will be larger. Compared with bulk ceramic, the grain size of the thin film structure is greatly reduced (usually from *μ*m to nm), and accompanied by the sharp reduction of the ferroelectric domains and the B-site ordered structure. In order to obtain a sufficiently large B-site ordered structure in the thin film and employ it to improve the tunability performance, the (Sc_1/2_Nb_1/2_) content shoud be higher.

In this work, we demonstrate a great improvement of dielectric tunable performance on the sol-gel-prepared Pb(Sc_1/2_Nb_1/2_)_0.9_(Mg_1/3_Nb_2/3_)_0.1_O_3_ (PSNMN) thin film via a newly proposed annealing method. As the as-prepared thin film was annealed under the help of an atmosphere-compensating-block (ACB) made from the proto-PSNMN gel, its dielectric tunability can be almost doubled from ~47% to ~80.0% at a low electric feild (~530 kV/cm), and its dielectric loss can be decreased by more than an order of magnitude from ~0.50 to ~0.037. Particularly, the PSNMN thin film annealed with ACB also exhibited an high thermally-stable dielectric tunability in an ultra-broad temperature range (>130 K). The Maxwell-Wagner (MW) effect generated by the interface between the PSNMN disordered matrix and the B-site nanoscale-ordered structure and the lower concentration of oxygen vacancy can be responsible for the significant performance improvement. The breakthrough on the dielectric tunable performance of A(B'_1/2_B”_1/2_)O_3_ ferroelectric thin film with a B-site nanoscale-ordered structure structure provides a new universal strategy to achieve utrahigh tunable performance, meanwhile it paves the way for a large-scale commercial applications of next-generation high performance ultraintegrated tunable thin-film-devices.

## 2. Results

### 2.1. Structure

**Figure 1:**
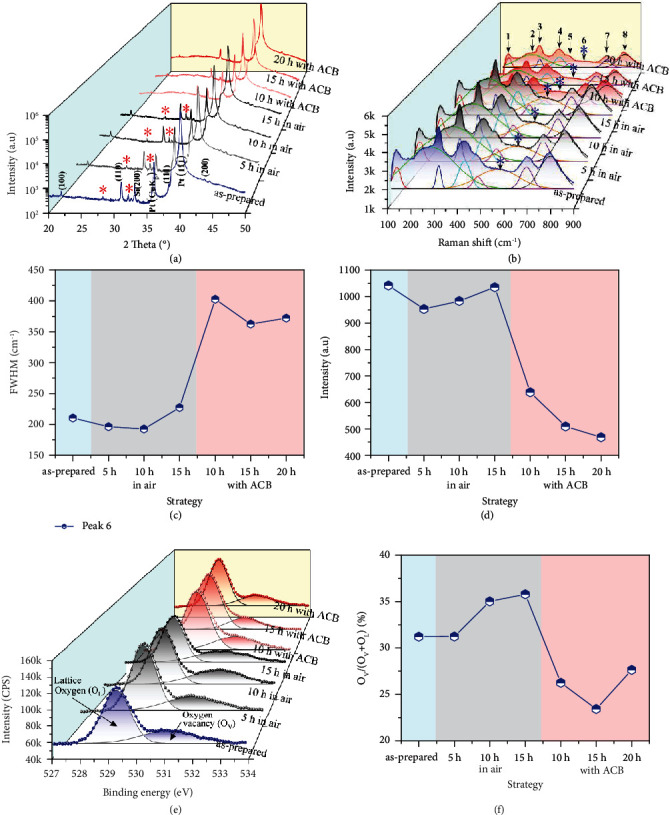
Spectroscopic characterization of PSNMN thin films. (a) XRD patterns. (b) Raman scattering spectra. (c) FWHMs and (d) Raman intensities of peak 6. (e) XPSs spectra of O 1 s. (f) O_v_/(O_v_ + O_L_)(%) calculated from (e).

Figure 1(a)) shows the X-ray diffraction (XRD) patterns of PSNMN thin films. All thin films exhibit a good crystallinity and a pure perovskite structure with a strong (111) orientation. Aside from the (111)-preferred diffraction peak, the (100) and (110) diffraction peaks are also visible. Compared with the thin film as prepared and the thin films annealed in air, the (110) peak of thin films annealed with ACB tends to weaken, but the (111) peak tends to strengthen. A small amount of impurities including pyrochlore phases (see red asterisks) can be observed on the patterns of both the as-prepared thin film (blue curve) and the thin films annealed in air (black curves). By contrast, impurities are invisible on the patterns of the thin films annealed with ACBs (red curves). In SEM images, these impurities can also be directly observed on thin film surfaces, as shown in Figure S1(b), S1(d), S1(f), and S1(h), where many white granular precipitates are clearly visible. However, the surface of thin films will become almost free of impurities as the ACBs were added, as shown in SEM images in Figure S1(j), S1(l), and S1(n). Furthermore, the grain size distribution of thin films will become narrower as the ACBs were added (see insets of Figure S1(b), S1(d), S1(f), S1(h), S1(j), S1(l), and S1(n)), and the columnar-like texture with (111) preferential orientation will also become more obvious, as shown in Figure S1(a), S1(c), S1(e), S1(g), S1(i), S1(k), and S1(m) [17–19]. Aforementioned results indicate that the ACBs can effectively promote the formation of the perovskite phase and improve the degree of orientation.

Figure 1(b)) shows the Raman scattering spectra of PSNMN thin films. Eight characteristic peaks can be fitted to each spectrum. Among these characteristic peaks, peak 6 (~530-580 cm^−1^; see blue asterisks) is the F_2g_ symmetric stretching mode and attributed to the Mg/Sc/Nb-O (B-site-O) stretching vibration modes. The appearance of the vibration modes suggests the existence of oxygen vacancies [20]. The full-width half maximum (FWHM) of peak 6 becomes wider (Figure 1(c)) as annealed with ACB, while the corresponding Raman intensity becomes weaker (Figure 1(d)), indicating a lower content of oxygen vacancies. To further confirm this, oxygen-related X-ray photoelectronic spectroscopy (O-related XPS) was used, as shown in Figure 1(e)). The split of two peaks were observed on each spectra. One peak with lower binding energy (~529.3 eV) is ascribed to the lattice oxygen (O_L_) and the other with higher binding energy (~531.2 eV) is arranged to the oxygen vacancy (O_V_) [21]. These XPS results are in consistent with the Raman analysis, which shows the PSNMN thin films annealed with ACB exhibit a lower content of oxygen vacancies. As shown in Figure 1(f)), the concentration of oxygen vacancies can be estimated by using the area ratio of O_V_/(O_V_ + O_L_). Especially for the thin film annealed with ACB for 15 h, the content of oxygen vacancies is the lowest (~23.4%), about 12.4% less than that of the thin film annealed in air for 15 h (the highest; ~35.8%). With the increase of the annealing time, the lead in the PSMN thin film will not be effectively compensated due to the loss of lead in the ACB atmosphere block. As a result, the content of oxygen vacancies will increase as the annealing time increases from 15 h to 20 h.

**Figure 2:**
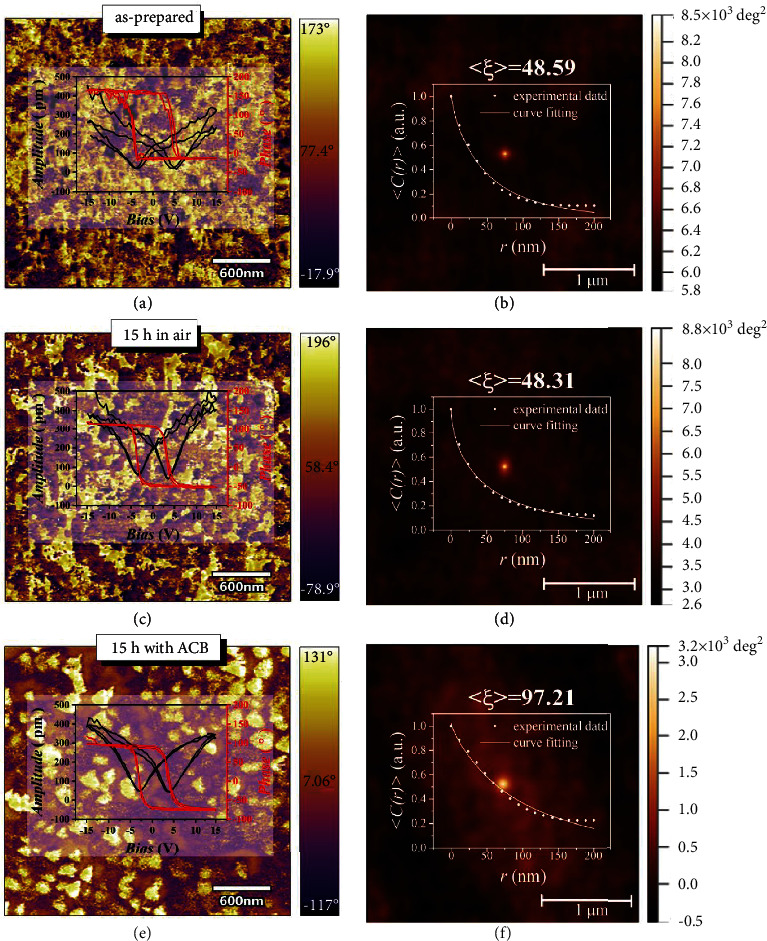
Vertical PFM images and corresponding autocorrelation images of PSNMN thin films. (a) and (b) as-prepared. (c) and (d) annealed in air for 15 h. (e) and (f) annealed with ACB for 15 h. Insets of (a), (c), and (e) butterfly curves of strain voltage response and loops of phase voltage response. Insets of (b), (d), and (f) <*C*(*r*)>.

Figure 2 shows the vertical PFM images of the as-prepared thin films, annealed for 15 h in air and with ACB. Some white island-like domains (average size: ~150 to 200 nm) with out-of-plane orientation can be observed for the thin film annealed with ACB for 15 h, however, they cannot be detected for the as-prepared thin films and annealed in air for 15 h, and only domains with irregular shape and random orientation are visible. The butterfly curve observed in the voltage strain response is becoming more complete as the ACB was added, as shown in the inset of Figures 2(a), 2(c), and 2(e), and the flipping degree of the corresponding P-E-like loop observed in the voltage phase response becomes larger, indicating a stronger piezoresponse. Particularly, the strain value and the flipping degree of the thin film annealed with ACB for 15 h are ~350 pm and ~180°, respectively.

In order to further study the characterization of domains in the above mentioned three thin films, the autocorrelation images revealing the polarization-distribution-pattern of polar nanoregions (PNRs) were investigated. Based on the PFM vertical images, the autocorrelation images (Figures 2(b), 2(d), and 2(f)) can be transformed by using the following relation [22–25]:

(1) $$\begin{array}{c} C\left(r_{1},r_{2}\right)=\underset{x,y}{\sum }D\left(x,y\right)D\left(x+r_{1},y+r_{2}\right), \end{array}$$

where *D*(*x*, *y*) represents the piezoresponse signal at position (*x*, *y*) in images. The negative or positive values of *C*(*r*_1_, *r*_2_) related to the probabilities of finding a region with antiparallel or parallel direction of the polarization after a shift on (*r*_1_, *r*_2_) from an arbitrary point in the original PFM images. The polarization radius is refered to the width of the central bright spot in the center of the autocorrelation images (Figures 2(b), 2(d), and 2(f)). Obviously, the polarization radius of the thin film annealed with the ACB was the biggest.

The short-range correlation length (*ξ*) representing the degree of polarization correlation can be calculated by using the average value of the autocorrelation function over in-plane directions, as follows:

(2) $$\begin{array}{c} \langle C\left(r\right)\rangle =\sigma^{2}\exp\left[-{\left(r/\langle \xi \rangle \right)}^{2h}\right], \end{array}$$

where *r* represents the position from the intermediate peak, and *h* (0 < h < 1) the roughness of the interface at the polarization point (the interface between PNRs). The higher the degree of polarization correlation, the larger PNRs and the greater the degree of order. According to the fitting results of the Equation (2) (insets of Figures 2(b), 2(d), and 2(f)), the *ξ* values of the three samples are ~48.6 nm, ~48.3 nm, and ~97.2 nm, respectively, indicating that the size of PNRs in thin film is increased largely and the corresponding order-degree is also enhanced as annealed with ACB.

**Figure 3:**
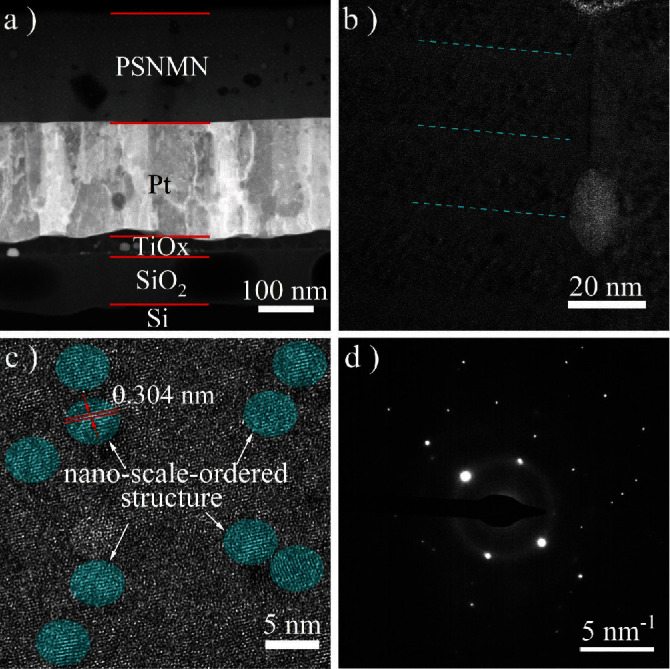
Cross-sectional TEM characterization of PSNMN thin film annealed with ACB for 15 h. (a) Full view cross-sectional HAADF-STEM image. (b) Enlarged view of HAADF-STEM image from PSNMN layer. (c) High resolution bright field TEM image. (d) SAED pattern.

Figure 3(a)) shows the full view of the cross-sectional HAADF-STEM image of PSNMN thin film annealed with ACB for 15 h. Each PSNMN layer (~19.2 nm) including many small bumps was detected and shown in the enlarged view of thin film (Figure 3(b)). These small bumps are more clearly visible in high-resolution bright field TEM mode (Figure 3(c)), and they can be ruled out as lead oxides based on the crystal-plane-spacing (~0.304 nm; see the cyan ellipse). According to previous research works [26], they could be named as B-site nanoscale-ordered structures. Due to the small size of these nanoscale-ordered structures (~5 nm), their diffracted electron signals are very weak and cannot be detected on the SAED pattern (Figure 3(d)). By comparasion, the B-site-ordered structures could be observed easily in the bulk ceramic due to the large size (>20 nm).

### 2.2. Dielectric Properties

**Figure 4:**
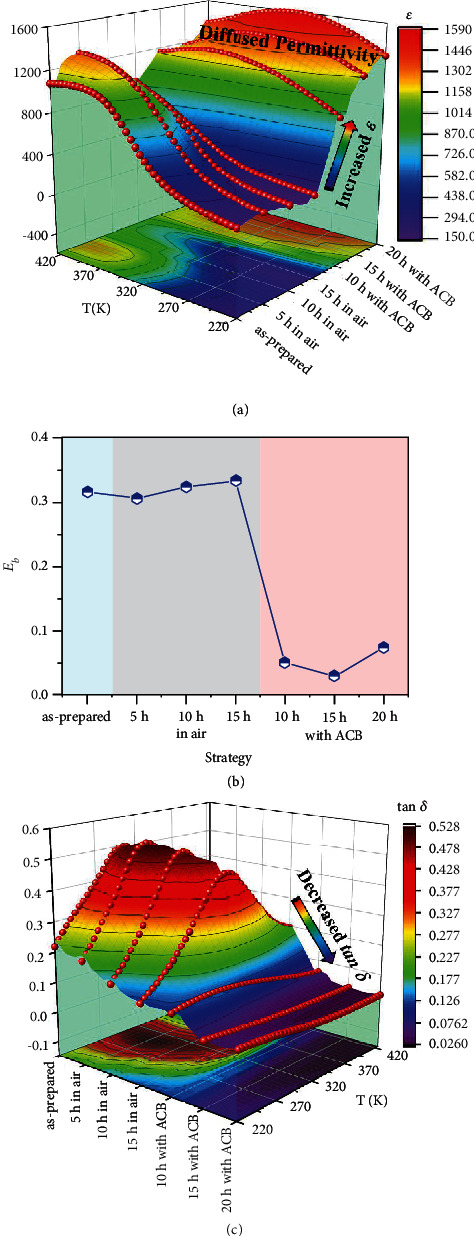
Temperature dependence of dielectric permittivity and dielectric loss of PSNMN thin films at 1 kHz. (a) *ε*(*T*). (b) *E*_*b*_ of PNRs. (c) tan *δ*(*T*).

Figure 4(a)) shows the temperature dependence of the dielectric permittivity (*ε*(*T*)) of PSNMN thin films at 1 kHz. The dielectric permittivity of PSNMN thin film as annealed with ACB is largely enhanced in the whole studied temperature range, especially in the range below room temperature (i.e. the low temperature regime). In the low temperature regime, the values of the *ε* of the as-prepared thin films and annealed in air are almost all less than 300. However, the maximum of the *ε* of the thin films annealed with ACB can almost reach ~1550, and the peaks of dielectric permittivity look more diffused compared with the as-prepared thin films and annealed in air. To uncover the dielectric performance of these thin films, we introduce the macroscopic phenomenological statistical model [27]. This model classifies individual dipoles into varies groups. Each have different dynamics and make distinctive contributions to the total dielectric permittivity [28]. An average potential-well-depth (*E*_b_), which is related to the size of PNRs, is used to characterize interactions between dipoles [29, 30]. A smaller *E*_b_ means more dipoles jumped from one temporal energy minimum to another under a stimulation of alternating current (AC) electrical field [27, 31]. According to the model, the *ε*(*T*) curve can be described by the following equation [32]:

(3) $$\begin{array}{c} \varepsilon \left(T\right)=\frac{\varepsilon_{1}}{1+b\exp\left(-\theta /T\right)}P_{1}\left(E_{b},T\right)+\varepsilon_{2}P_{2}\left(E_{b},T\right), \end{array}$$

where *ε*_1_, *ε*_2_, *b*, and *θ* are constants at a given frequency, and *T* is the absolute temperature. Equation (3) well fits all *ε*(T) curves, and the fitting parameters are summarized in Table S1 [33]. Compared with the as-prepared thin films and the annealed thin films in air, except for the *ε*_2_, all the parameters decrease for the sample as annealed with ACB. The sharply decreased Eb (Figure 4(b)) indicates more dipoles can be activated, and a higher degree of relaxor diffusion is developed. In addition, the thermal stability of the dielectric permittivity of the thin film annealed with ACB is also significantly improved with the enhanced dielectric permittivity and the increased degree of relaxor diffusion.

Figure 4(c) shows the temperature dependence of the dielectric loss (tan *δ*(*T*)) of PSNMN thin films at 1 kHz. Different to the great enhancement in the dielectric permittivity around room temperature, the dielectric loss of the thin film is sharply decreased from ~0.50 (the as-prepared) to ~0.037 (the annealed with ACB for 15 h). It is almost reduced by an order of magnitude. Moreover, the value of the dielectric loss will be further reduced as the temperature increases in the studied range.

### 2.3. Dielectric Tunabilities

The dielectric tunability (*η*) is defined in Equation (4) using the dielectric permittivity at zero (*ε*(0)) and a certain DC electric field (*ε*(*E*)). The figure-of-merit (FOM) is defined in Equation (5) using dielectric loss at a certain electric field (tan *δ*(*E*)) instead of the constant (tan *δ*(0)) at zero electric field [13, 14, 34, 35]:

(4) $$\begin{array}{c} \eta \left(\%\right)=\left(\varepsilon \left(0\right)-\varepsilon \left(E\right)\right)/\varepsilon \left(0\right)\times 100, \end{array}$$

(5) $$\begin{array}{c} \text{FOM}\left(E\right)=\eta \left(E\right)/\tan\;\delta \left(E\right). \end{array}$$

**Figure 5:**
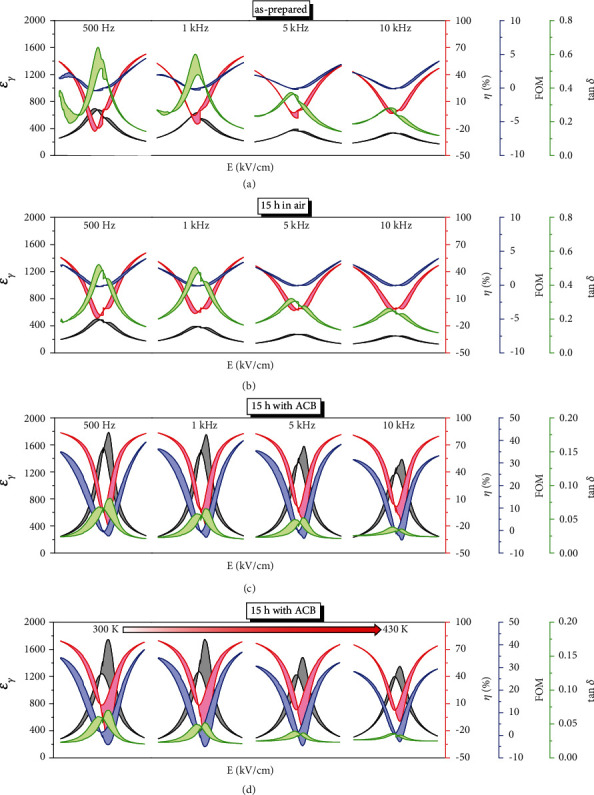
Dielectric tunable performance of PSNMN thin films. (a), (b), and (c) the *ε*_*γ*_(*E*), *η*(*E*), FOM(*E*) and tan *δ*(*E*) of the as-prepared, annealed in air for 15 h and annealed with ACB for 15 h at 500 Hz, 1 kHz, 5 kHz, and 10 kHz and at room temperature. (d) The *ε*_*γ*_(*E*), *η*(*E*), FOM(*E*), and tan *δ*(*E*) of the PSNMN thin film annealed with ACB for 15 h at 300 K, 340 K, 390 K, and 430 K.

Figure 5 and Figure S2 show the relationship of DC bias via dielectric permittivity (*ε*_*γ*_(*E*)), the dielectric tunability (*η*(*E*)), the figure-of-merit (FOM(*E*)), and the dielectric loss (tan *δ*(*E*)) of the PSNMN thin films at 500 Hz, 1 kHz, 5 kHz, and 10 kHz at room temperature. The *ε*_*γ*_(*E*), tan*δ*(*E*) curves exhibit bell shapes, while *η*(*E*) and FOM(*E*) curves present petal shapes. These phenomena reveal a nonlinear effect driven by an electric field, which is mainly related to the activities of the domain-wall and the cluster interphase boundaries, the reorientated dipolar moments of PNRs, and the polarized lattice phonon. The dielectric tunability of as-prepared PSNMN thin films, annealed in air for 5 h, 10 h, and 15 h, annealed with ACB for 10 h, 15 h, and 20 h at 530 kV/cm and 10 kHz is ~47.0%, ~ 46.5%, ~ 48.5%, ~ 46.8%, ~ 76.4%, ~ 80.0%, and 77.3%, respectively, and the FOM is ~3.99, ~ 4.39, ~ 4.28, ~ 3.94, ~ 33.0, ~ 33.2, and~ 33.1, respectively. As the direction of applied DC electric field is reversed to -530 kV/cm, the dielectric tunability of PSNMN thin films is ~43.3%, ~ 43.0%, ~ 45.4%, ~ 45.4%, ~ 76.7%, ~ 80.8%, and~ 75.0%, respectively, and the FOM is ~2.3, ~ 2.6, ~ 2.8, ~ 3.0, ~ 25.7, ~ 31.5, and~ 29.2, respectively.

**Figure 6:**
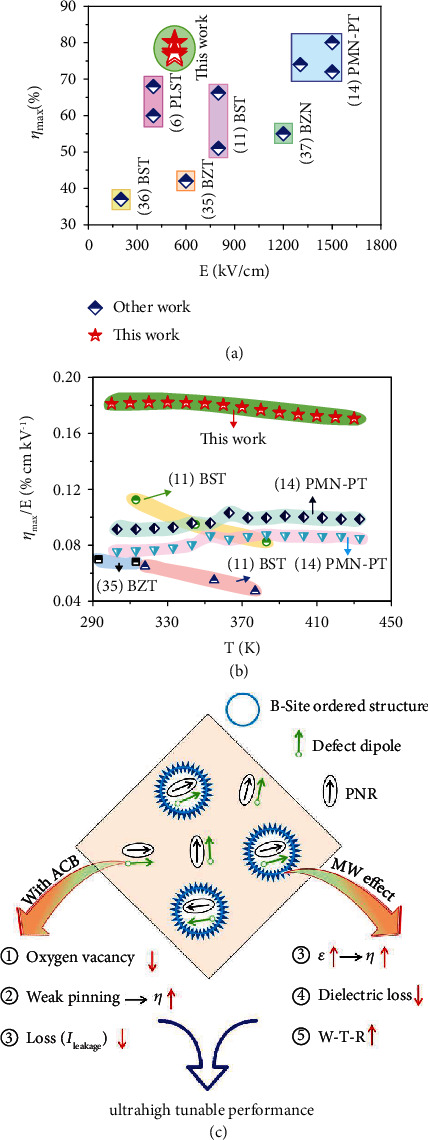
Comparsion of dielectric tunable performance in perovskite thin films. (a) *η*_max_ achieved by the applied *E*. (b) *η*_max_/*E* at the studied temperature range. (c) Mechanism for the significant dielectric tunable performance.

It can be seen that the dielectric tunability and the FOM of the thin films can be significantly enhanced after annealed with ACB, and the former is almost doubled and the latter is increased by an order of magnitude. Particularly, the thin film annealed with ACB for 15 h exhibits the best dielectric tunability and FOM, and they all are almost frequency-independent within the studied frequency range (Figure 5(c)). By contrast, the dielectric tunability of the as-prepared thin films and the thin films annealed in air varies drastically with frequency although the FOMs look frequency independent. For example, the dielectric tunabilities of as-prepared thin film at 500 Hz, 1 kHz, 5 kHz, and 10 kHz are 62.7%, 60.4%, 49.7%, and 47.0%, respectively. Moreover, the dielectric tunability of the thin film annealed with ACB for 15 h is also almost temperature independent. As shown in Figure 5(d), as the temperature increases from 300 K to 430 K, the variation (the maximum fluctuation to the middle value) of the dielectric tunability at 430 kV/cm is less than 4%. For the FOM, the variation is less than 15%, which is acceptable for practical application. These results indicate that the dielectric tunability, the FOM, the thermal stability, and the frequency stability of thin film can be significantly improved after annealing with ACB.  Figure 6 compares the dielectric tunable performance of reported perovskite thin films with the PSNMN thin films annealed with ACB [6, 11, 14, 36–38]. It can be seen that a higher *η*_max_ is achived by the PSNMN thin films annealed with ACB at a lower applied *E*, as shown in Figure 6(a)). Moreover, the PSNMN thin films annealed with ACB exhibit a higher *η*_max_/*E* ~ 0.18%cmkV^−1^ at the studied temperature range as well as a higher thermal stability, as show in Figure 6(b)).

## 3. Discussion

Previous researches [16, 29, 35, 39–42] have suggested that a high dielectric tunability is associated to both the intrinsic lattice phonon polarization and the extrinsic polarization. The higher dielectric tunability of the thin films annealed with ACB may be related to the higher purity of perovskite structure (see the XRD analysis results) because the impurities (pyrochlore phases, etc.) usually deteriorate the lattice phonon polarization [16, 35, 39]. In addition to the intrinsic lattice phonon polarization, the higher dielectric tunability of the thin films annealed with ACB may also be related to the contribution of the extrinsic polarization, which mainly includes the reorientation of PNRs and the activity of the cluster interphase boundaries or the domain-wall motions [39]. Ususally, the formation of the defects such as oxygen vacancies is not conductive to the domain-wall motions or the reorientation of PNRs because of the pinning effect. In view of the analysis results of Raman spectra and XPS spectra, we have learned that a lower content of oxygen vacancies is obtained on the thin films annealed with ACB. Therefore, the pinning effect of oxygen vacancies in the thin films annealed with ACB should be the weakest.

To verify the proposed hypothesis, an imprinting experiment based on the PFM vertical phase (out-of-plane) mode was carried out. As shown in Figure S3, the left and right rectangular areas of PSNMN thin films were poled by using a DC bias of –15 V and+ 15 V at first, respectively. Subsequently, the whole studied area was scanned under a 0 V DC bias for the first cycle. When waiting for 30 min, the second scanning cycle was carried out. After waiting for another 30 min, the third scaning cycle was executed. As time went on, the rectangular areas of the thin films annealed with ACB for 15 h gradually blurred, as shown in Figure S3(c), indicating a weak imprinting effect. By contrast, the rectangular areas of the as-prepared thin film and the thin film annealed in air for 15 h are still visible clearly, as shown in Figure S3(a) and S3(b), revealing a strong imprinting effect. It has been well known that the imprinting effect is mainly originated from the pinning effect of defect. Therefore, we can conclude that the imprinting effect of studied thin films is mainly ascribed to the pinning effect of oxygen vacancies, and the thin film annealed with ACB for 15 h shows the weakest pinning effect of oxygen vacancies. As a result, the dielectric tunability will be significantly improved by the extrinsic contribution, as shown in Figure 5 and Figure S2.

Early classical dielectric theory [43] has pointed out that dielectric loss is generally composed by the conductivity loss, the polarization loss, ionization loss, etc. A higher content of oxygen vacancies will not only increase the conductivity loss but also aggravate the polarization loss. As shown in Figure S4(a), the P-E loop was bloated obviously from the thin films annealed in air for 15 h due to the highest oxygen vacancies concentration (see Figure 1(f)), indicating the existence of a large leakage current. By contrast, the P-E loops were all slim for the thin films annealed with ACB, indicating the appearance of a smaller leakage current. Moreover, the coercivity field (*E*_c_) of thin film has also been greatly reduced as annealed with ACB, and the inversion current of domains (*I*_domain_) increases greatly, as shown in the I-V curves (Figure S4(b)). Especially, the value of *I*_domain_ has been increased from ~12000 mA/cm^2^ (the thin film annealed in air for 15 h) to ~18000 mA/cm^2^ (the thin film annealed with ACB for 15 h). The enhancement of *I*_domain_ not only indicates a higher mobility of domains but also reveals a weaker pinning effect of defects (oxygen vacancies). Therefore, we can conclude that the lower dielectric loss of thin films annealed with ACB may be mainly related to the lower content of oxygen vacancies. It should be noted that the dielectric loss of thin films can be further reduced at a millimeter-wave-band as reported in previous researches [44, 45]. Furthermore, simulation results (supplementary Figure S5) also confirm that the device based on our thin films annealed with ACB possesses lower insertion loss (less than dB = 0.328) and very high phase-shift-ability (up to 170°) at a microwave band (~36 GHz), indicating an excellent dielectric tunable performance. Moreover, the material type and geometry of the electrode have a certain impact on the dielectric loss. Therefore, further research should be carried out to further optimize the overall tunable performance of the device.

The Maxwell-Wagner polarization mechanism [46–52] was widely employed to explain the high dielectric permittivity observed in single-phase ceramics, single crystals, and composites. Generally, the large dielectric permittivity resulted from the Maxwell-Wagner relaxation occurring at grain boundaries and phase boundaries. As shown in the cross-sectional TEM images (Figures 3(b) and 3(c)), there must be a lot of interfaces between the nanoscale-ordered superlattice structures and the disordered matrix. So, the large enhancement (Figure 4(a)) of the dielectric permittivity of thin films annealed with ACB may be related to the Maxwell-Wagner effect based on the relaxation occurring at the ordered-disordered interefaces. It should be noted that an underlying Maxwell-Wagner mechanism was also reported to be responsible for dielectric permittivity enhancement in some superlattices published previously [53]. Some research works have also revealed that the dielectric loss can be reduced based on the nanoscale-ordered structures, although the underlying mechanism is not clear [54]. Therefore, the reduced dielectric loss of thin films annealed with ACB may be related to the Maxwell-Wagner effect, in addition to the lower content of oxygen vacancies. For this case, further research is needed in the future.

We have learned that the thermal stability of the dielectric permittivity of thin film annealed with ACB is related to both the enhanced dielectric permittivity and the increased degree of relaxor diffusion, as shown in results section. Therefore, the Maxwell-Wagner effect may aslo be responsible for the high thermal stability of the dielectric permittivities of thin films annealed with ACB, and naturally for the high thermal stability of the dielectric tunabilities and the FOMs. To summarize, a mechanism diagram is suggested for the roles of the ACB and the MW effect on the significant dielectric tunable performance, as shown in Figure 6(c).

The high tunability, the low dielectric loss and the high thermal stability of the PSNMN thin films annealed with ACB are comparable to those of highly oriented PMN-PT thin films deposited on the expensive Nb-doped STO single crystal substrates. Particulary, the applied DC electric field (~530 kV/cm) of the former is far below than that (~1500 kV/cm) of the latter. It is well-known that the lower the electric field means the higher the safety and fatigue.

It must be pointed out that a moderate dielectric permittivity is also needed for tunable materials in practical applications, in addtion to the high tunability, the low dielectric loss, and the high thermal stability. Some feasible strategies could be suggested, such as decreasing crystallization temperature, controlling the ratio of (Sc_1/2_/Nb_1/2_) to (Mg_1/3_Nb_2/3_), and adjusting the orientation of thin film. Furthermore, research work on the tunable performance at a millimeter-wave-band needs to be carried out.

Moreover, the energy storage performance can also be significantly improved as annealed with ACB, as shown in Figure S6. The recoverable energy density (*W*_energy_; at 1990 kV/cm) was enhanced from 22.3 J/cm^3^ of as-prepared thin film to 27.2 J/cm^3^ of thin film annealed with ACB for 15 h, and the efficiency (*η*_energy_) was increased from 47.0% to 75.1% [55–60]. Particularly, the thermal stability was also significantly improved, and the variation of the maximum fluctuation to the middle value of *η*_energy_ at ~1700 kV/cm is less than 2% in the temperature range of 228 K to 423 K. The energy storage performance is comparable to the state-of-art lead-involved thin films such as the low-temperature-poling awakened PLZST (31.2 J/cm^3^ with a *η*_energy_ ~ 58% at ~2000 kV/cm) [55] and also the best lead-free compounds such as the solution-processed ferroelectric terpolymer nanocomposites (20.3 J/cm^3^ with a *η*_energy_ ~ 77% at 6500 kV/cm) [61]. Therefore, annealing with ACB can also be regarded as a new strategy to improve the energy storagy performance.

## 4. Conclusion

The dielectric tunability performance of the sol-gel-prepared PSNMN thin film is significantly improved after annealed with ACB. The dielectric tunability is largely increased (from ~47% to ~80.0% at ~530 kV/cm), and the dielectric loss is sharply decreased (from ~0.50 to ~0.037). Particularly, a high thermally-stable dielectric tunability is realized in an ultrabroad temperature range (>130 K). The significant improvement in dielectric tunablility may be related to the Maxwell-Wagner effect generated by the interface between the PSNMN disordered matrix and the B-site nanoscale-ordered structure and the lower concentration of oxygen vacancy. This work provides a new strategy to achieve highly tunable ferroelectric thin films and paves the way for the commercial application of next-generation tunable devices.

## 5. Materials and Methods

### 5.1. Fabrication of PSNMN Thin Films

**Figure 7:**
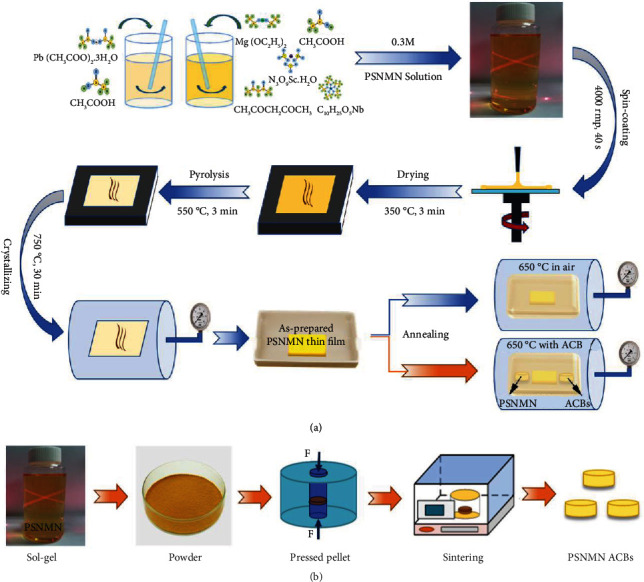
Flow charts of sample preparation. (a) Pb(Sc_0.5_Nb_0.5_)_0.9_(Mg_1/3_Nb_2/3_)_0.1_O_3_ (PSNMN) thin films. (b) PSNMN atmosphere-compensating-blocks.

The sol-gel method was used to fabricate PSNMN thin films, as shown in Figure 7(a). Lead acetate trihydrate with 20% excess lead was dissolved in glacial acetic acid (Pb solution). Meanwhile, magnesium ethoxide with 20% excess magnesium, niobium ethoxide, and scandium nitrate hydrate were dropped into a solution of glacial acetic acid and acetylacetone (Sc/Nb/Mg solution). Subsequently, the Pb solution and the Sc/Nb/Mg solution were mixed and stirred for 30 min and the final concentration of the PSNMN precursor solution was calibrated to be 0.3 M by using glacial acetic acid. After aging for 24 h, the PSNMN precursor solution was spin-coated onto the Pt(111)/TiO_x_/SiO_2_/Si substrate at 4000 rpm for 40 s, and the resulted wet film was dried at 350°C for 3 min on a hotplate, and then it was pyrolyzed at 550°C for 3 min on another hotplate. The above mentioned procedures were repeated for 12 times. Finally, the PSNMN thin film was crystallized at 750°C for 30 min in a tube furnace and in air.

The as-prepared PSNMN thin film was cut into smaller pieces, and three pieces of them were annealed at 650°C in a crucible with a lid and in air, and the annealing time for them was 5 h, 10 h, and 15 h, respectively. Different from these three pieces, another three pieces were annealed under the help of an atmosphere-compensating-block (ACB) made from the PSNMN gel, and the annealing time of them was 10 h, 15 h, and 20 h, respectively. For convenience, these annealed samples were named as 5 h in air, 10 h in air, 15 h in air, 10 h with ACB, 15 h with ACB, and 20 h with ACB, respectively. The thicknesses of thin films (~ 217 to ~257 nm) were determined from the cross-sectional SEM images (Figure S1(a), S1(c), S1(e), S1(g), S1(i), S1(k), and S1(m)), depending on the preparation conditions, which also determined the surface SEM images (Figure S1(b), S1(d), S1(f), S1(h), S1(j), S1(l) and S1(n)) of the thin films.

### 5.2. Fabrication of Atmosphere-Compensating-Blocks

The conventional solid-state reaction method was used to prepare the ACB, as shown in Figure 7(b). Firstly, the PSNMN precursor solution was poured into a beaker that was cleaned in advance, and then it was stirred while drying at 150°C on a hotplate. After the precursor solution was dried thoroughly, the resulted PSNMN powder was pressed into pellets (diameter~ 5 mm, and thickness~ 1 mm). Subsequently, they were sintered at 1100°C for 2 h at a 3°C/min heating rate, and then cooled to 800°C at a 3°C/min cooling rate, and finally cooled naturally to room temperature.

### 5.3. Characterization

X-ray diffraction (XRD; Rigaku 9 kW Smart Lab, Tokyo, Japan) was employed to differentiate the phase structures of PSNMN thin films. Raman spectrometer (Horiba HR800, *λ* = 325 nm) was employed to collect the Raman scattering spectra. Field emission scanning electron microscopy (FE-SEM; ZEISS, Sigma 500, Germany) was used to observe the cross-sectional and surface morphologies of the thin films. The X-ray photoelectron spectrometer (XPS; PHI 5000 VersaProbe III, Ulvac-Phi, Japan) was employed to identify the oxygen vacancy. Piezoresponse force microscope (PFM; Bruker Multimode with a Nanoscope V Controller, Germany) was used to study the surface feature of thin films. Transmission electron microscope (TEM; Talos F200X, Thermo-Fisher, USA) was used to investigate the microstructure of thin films. For the electrical property measurements, 90^∗^90 *μ*m square Cr/Au top electrodes were deposited by the RF magnetron sputtering method using a stainless steel shadow mask. The dielectric tunability, dielectric loss, and dielectric permittivity were measured by an impedance analyzer (Agilent E4980A, the United States), and the perturbation voltage Vac is set to 100 mV. P-E loops and corresponding I-V curves were measured by a ferroelectricity tester (Precision Premier II, Radiant Technologies Inc., Alpharetta, GA).

## Data Availability

All data [XRD, Raman, dielectric permittivity, loss, tunability, SEM, TEM, PFM, XPS] used to support the findings of this study are included within the article. All data used to support the findings of this study may be released upon application to Prof. Biaolin Peng, who can be contacted at pengbl8@126.com.

## Supplementary Materials

Supplementary Materials Figure S1. Cross-sectional and corresponding surface SEM images of PSNMN thin films. (a) and (b) the as-prepared. (c), (d), (e), (f), (g), and (h) annealed in air. (i), (j), (k), (l), (m) and (n) annealed with ACB. Insets: the statistical distribution diagram of the average grain size. Figure S2. The *εγ*(E), *η*(E), FOM(E), and tan *δ*(E) of the PSNMN thin films. Figure S3. PFM vertical phase images of the PSNMN thin films. (a) as-prepared. (b) annealed in air for 15 h. (c) annealed with ACB for 15 h. The left and right rectangular areas of samples were poled by using a DC bias of –15 V and + 15 V at first, respectively. Subsequently, the whole studied area was scanned under a 0 V DC bias for the first cycle. Waiting for 30 min, the second scanning cycle was carried out, and then waiting for another 30 min, the third scaning cycle was done. Figure S4. Ferroelectric performance of the PSNMN thin films. (a) P-E loops. (b) I-E curves. Figure S5. Simulating results of high frequency structure simulator based on the coplanar transmission line method. (a) Phase shift degree and phase shift function. (b) Insertion loss. (c) HFSS simulation. The simulation was done by using the high frequency structure simulation software (HFSS). The periodic loading form of distributed ferroelectric thin film capacitors were employed to design and simulate the phase shifter. To form a composite transmission line, the basic structure is consisted by a series of controllable variable PSNMN thin-film-capacitors periodically loaded on the coplanar waveguide (CPW) high resistance transmission line. By applying a DC bias to the PSNMN thin film capacitors, the dielectric constant of the thin film is changed, and the loading capacitance is adjusted to cause phase change. The loading capacitance is designed to be in the form of interdigital capacitance (IDC), as shown in the upper part of Figure S5(c). For the IDC, the main dimensions are the following: LCPW = 1 mm, G = 60 *μ*m, S = 100 *μ*m, T = 100 *μ*m, LIDC = 200 *μ*m, WIDC = 215 *μ*m, g = 110 *μ*m, WD = 5 *μ*m, and g1 = g2 = 10 *μ*m. The bias circuit in this phase shifter is composed of two quarter wavelength high and low impedance transmission lines, as shown in the lower part of Figure S5(c). Figure S6. Energy storage performance of PSNMN thin films at 10 kHz. (a) Wenergy(E). (b) ŋ(E). (c) Wloss(T). (d) ŋ(T). Table S1. Parameters fitting results of PSNMN thin films by using Equation (3).
