# Supplementary material for: B-Site Nanoscale-Ordered Structure Enables Ultra-High Tunable Performance
Source: Research (Wash D C). 2022 Oct 26;2022:9764976. doi: 10.34133/2022/9764976 (PMC9639443; doi:10.34133/2022/9764976)
Supplement: Supplementary Materials — Figure S1. Cross-sectional and corresponding surface SEM images of PSNMN thin films. (a) and (b) the as-prepared. (c), (d), (e), (f), (g), and (h) annealed in air. (i), (j), (k), (l), (m) and (n) annealed with ACB. Insets: the statistical distribution diagram of the average grain size. Figure S2. The εγ(E), η(E), FOM(E), and tan δ(E) of the PSNMN thin films. Figure S3. PFM vertical phase images of the PSNMN thin films. (a) as-prepared. (b) annealed in air for 15 h. (c) annealed with ACB for 15 h. The left and right rectangular areas of samples were poled by using a DC bias of –15 V and + 15 V at first, respectively. Subsequently, the whole studied area was scanned under a 0 V DC bias for the first cycle. Waiting for 30 min, the second scanning cycle was carried out, and then waiting for another 30 min, the third scaning cycle was done. Figure S4. Ferroelectric performance of the PSNMN thin films. (a) P-E loops. (b) I-E curves. Figure S5. Simulating results of high frequency structure simulator based on the coplanar transmission line method. (a) Phase shift degree and phase shift function. (b) Insertion loss. (c) HFSS simulation. The simulation was done by using the high frequency structure simulation software (HFSS). The periodic loading form of distributed ferroelectric thin film capacitors were employed to design and simulate the phase shifter. To form a composite transmission line, the basic structure is consisted by a series of controllable variable PSNMN thin-film-capacitors periodically loaded on the coplanar waveguide (CPW) high resistance transmission line. By applying a DC bias to the PSNMN thin film capacitors, the dielectric constant of the thin film is changed, and the loading capacitance is adjusted to cause phase change. The loading capacitance is designed to be in the form of interdigital capacitance (IDC), as shown in the upper part of Figure S5(c). For the IDC, the main dimensions are the following: LCPW = 1 mm, G = 60 μm, S = 100 μm, T = 100 μm, LI [file 9764976.f1.zip › Supplementary materials.doc]

**Supplementary Materials for**

**B-site nanoscale-ordered structure enables ultra-high tunable performance**

Biaolin Peng^1,2^*^§^, Qiuping Lu^1,2§^, Yi-Chi Wang^2^, Jing-Feng Li^3^*, Qi Zhang^4,5^, Haitao Huang^6^, Laijun Liu^7^, Chao Li,^8^ Limei Zheng^9^*, Zhong Lin Wang^1,2,10^*

*Correspondence to:

[pengbL8@126.com](mailto:pengbL8@126.com),

[jingfeng@mail.tsinghua.edu.cn](mailto:jingfeng@mail.tsinghua.edu.cn),

[zhenglm@sdu.edu.cn](mailto:zhenglm@sdu.edu.cn),

[zhong.wang@mse.gatech.edu](mailto:zhong.wang@mse.gatech.edu),

§These authors contributed equally to this work.

**This file includes:**

**Fig. S1** to **Fig. S6 and Table S1**

**Captions of Figures and Tables**

**Fig. S1.** **Cross-sectional and corresponding surface SEM images of PSNMN thin films.** a) and b) the as-prepared. c), d), e), f), g) and h) annealed in air. i), j), k), l), m) and n) annealed with ACB. Insets: the statistical distribution diagram of the average grain size.

**Fig. S2.** **The *ε*_γ_(*E*), *η*(*E*), FOM(*E*) and *tan δ*(*E*) of the PSNMN thin films.**

**Fig. S3.** **PFM vertical phase images of the PSNMN thin films.** a) as-prepared. b) annealed in air for 15 h. c) annealed with ACB for 15 h. The left and right rectangular areas of samples were poled by using a dc bias of –15 V and +15 V at first, respectively. Subsequently, the whole studied area was scanned under a 0 V dc bias for the first cycle. Waiting for 30 min, the second scanning cycle was carried out, and then waiting for another 30 min, the third scaning cycle was done.

**Fig. S4. Ferroelectric performance of the PSNMN thin films.** a) *P-E* loops. b) *I-E* curves.

**Fig. S5. Simulating results of high frequency structure simulator based on the coplanar transmission line method.**  a) Phase shift degree and phase shift function.  b) Insertion loss. c) HFSS simulation. The simulation was done by using the high frequency structure simulation software (HFSS). The periodic loading form of distributed ferroelectric thin film capacitors were employed to design and simulate the phase shifter. To form a composite transmission line, the basic structure is consisted by a series of controllable variable PSNMN thin-film-capacitors periodically loaded on the coplanar waveguide (CPW) high resistance transmission line. By applying a DC bias to the PSNMN thin film capacitors, the dielectric constant of the thin film is changed, and the loading capacitance is adjusted to cause phase change. The loading capacitance is designed to be in the form of interdigital capacitance (IDC), as shown in the upper part of Fig. S5c. For the IDC, the main dimensions are the following: L_CPW_ = 1 mm, G = 60 μm, S = 100 μm, T = 100 μm, L_IDC_ = 200 μm, W_IDC_ = 215 μm, g = 110 μm, W_D_ = 5 μm and g_1_ = g_2_ = 10 μm. The bias circuit in this phase shifter is composed of two quarter wavelength high and low impedance transmission lines, as shown in the lower part of Fig. S5c.

**Fig. S6. Energy storage performance of PSNMN thin films at 10 kHz.** a) *W_energy_*(*E*). b) *ŋ*(*E*)*.* c) *W_loss_*(*T*)*.* d) *ŋ*(*T*).

**Table S1.** **Parameters fitting results of PSNMN thin films by using equation (3).**

**Fig. S1**

**Fig. S2**

**Fig. S3**

**Fig. S4**

**Fig. S5**

**Fig. S6**

**Table S1**

| **parameter** | **as-prepared** | **5 h**  **in air** | **10 h**  **in air** | **15 h**  **in air** | **10 h with ACB** | **15 h with ACB** | **20 h with ACB** |
| --- | --- | --- | --- | --- | --- | --- | --- |
| ***ε_1_*** | 1.20 10^7^ | 1.11 10^7^ | 1.30 10^7^ | 1.01 10^7^ | 3.58 10^3^ | 2.35 10^3^ | 8.17 10^3^ |
| ***E_b_* (eV)** | 0.31605 | 0.30608 | 0.32425 | 0.33335 | 0.05111 | 0.03024 | 0.07469 |
| ***ε_2_*** | 217.65 | 241.82 | 212.76 | 212.89 | 375.81 | 500.02 | 951.34 |
| ***b*** | 340890.58 | 59489.35 | 335540.00 | 6934.41 | 114.51 | 265.94 | 180.51 |
| ***θ* (K)** | 4532.84 | 3812.02 | 4525.71 | 3098.76 | 2209.73 | 2634.29 | 1873.43 |
